# Fatal Coronavirus Disease 2019-associated Pulmonary Aspergillosis; A Report of Two Cases and Review of the Literature

**DOI:** 10.1016/j.idcr.2020.e00935

**Published:** 2020-08-22

**Authors:** Shiema Abdalla, Muna A. Almaslamani, Samar M. Hashim, Abdulsalam S. Ibrahim, Ali S. Omrani

**Affiliations:** a Communicable Diseases Center, Hamad Medical Corporation, Doha, Qatar; b Communicable Diseases Center and Division of Infectious Diseases, Hamad Medical Corporation, Doha, Qatar; c Division of Critical Care, Hamad Medical Corporation

**Keywords:** COVID-19, aspergillosis, ICU, viral pneumonia

## Abstract

Coronavirus Disease 2019 (COVID-19)-associated pulmonary aspergillosis is an emerging entity. We report two fatal cases of putative COVID-19-associated pulmonary aspergillosis. Both cases were diagnosed on the basis of respiratory tract cultures yielding Aspergillus species and otherwise unexplained clinical and radiological deterioration. Existing published literature on COVID-19-associated pulmonary aspergillosis indicate poor outcomes and high mortality. CAPA should be considered in patients with critical COVID-19 who have unexplained progressive respiratory failure despite optimized supportive care. Diagnostic work-up should be initiated as early as possible and should ideally include fungal cultures, galactomannan detection and Aspergillus PCR on tracheal aspirates or broncho-alveolar lavage fluid. Empiric systemic antifungal therapy may be justified in selected cases, pending diagnostic work up results. Large, multi-center studies are required to further understand the pathogenesis of invasive aspergillosis in COVID-19, and the optimal diagnostic and treatment strategies.

## Background

Worldwide, more than 10 million confirmed infections with the novel Severe Acute Respiratory Syndrome Coronavirus 2 (SARS-CoV-2), the cause of Coronavirus Disease 2019 (COVID-19), have been reported. [1] Though the majority of COVID-19 case are mild, approximately 5-10% of affected individuals may require invasive ventilation in an intensive care unit (ICU). [2] Invasive pulmonary aspergillosis is a recognized complication of severe influenza and Severe Acute Respiratory Syndrome (SARS). [[3], [4], [5]] COVID-19-associated pulmonary aspergillosis (CAPA) is an emerging entity wherein *Aspergillus species* cause invasive pulmonary disease in patients with critical COVID-19. [6] We herein describe two fatal cases of CAPA from Qatar and review the available literature on the subject.

## Case Reports

The first patient was a 58-year-old man with known history of diabetic nephropathy, systemic hypertension, hyperlipidemia, and chronic hepatitis B infection. He presented with a 5-day history of fever, sore throat, cough and dyspnea. His baseline assessment showed evidence of severe respiratory distress (heart rate 122 beats per minute, respiratory rate 33 breaths per minutes and oxygen saturation 90% on high flow nasal oxygen). His baseline blood investigations showed arterial partial pressure of oxygen (PaO2) of 30 mmHg, peripheral lymphocyte count of 3.8 × 10^9^ cells per L, and C reactive protein (CRP) of 335.2 mg/L. Patchy confluent airspace opacification was evident on his chest x-ray. He was transferred to ICU for invasive mechanical ventilation. On the second day, he developed rapidly progressive renal failure, hyperkalemia, and mixed respiratory and metabolic acidosis. Renal replacement therapy with sustained low-efficiency dialysis was started. A nasopharyngeal swab PCR for SARS-CoV-2 was positive. His treatment included hydroxychloroquine, azithromycin, lopinavir-ritonavir, interferon alfa-2a, broad-spectrum antibacterial therapy, a single dose of tocilizumab 400 mg on day 2, and methylprednisolone 80 mg daily from day 3 onwards. A chest x-ray repeated on day 5 showed some regression of the baseline pulmonary opacities. On day 6, a lower respiratory tract culture was reported to yield a growth of *Aspergillus niger* and *Candida albicans.* On day 11, the patient’s respiratory status deteriorated and his follow up chest x-ray showed worsening bilateral infiltrates. No bacterial pathogens were isolated from cultures of the lower respiratory tract or any other sites. On day 14, the lack of clinical improvement lead to a diagnosis of putative CAPA and combination antifungal therapy with anidulafungin and liposomal amphotericin was started. Unfortunately, the patient’s condition continued to deteriorate and he passed away on day 15 of hospitalization.

The second case was a 74-year-old man with no pre-existing chronic medical conditions. He presented with an 8-day history of fever, sore throat, productive cough and dyspnea. His baseline assessment showed oral temperature of 38 °C, heart rate of 102 beats per minute, respiratory rate of 23 per minute and SpO2 of 94% on ambient room air. Chest x-ray showed extensive bilateral pulmonary infiltrates. The next day, he developed progressive respiratory failure (PaO2 48 mmHg) and required intubation and invasive mechanical ventilation. Blood investigations at the time showed peripheral lymphocyte count of 0.6 × 10^9^/L, and CRP 90.1 mg/L. SARS-CoV-2 PCR on a nasopharyngeal swab was positive. He was started on hydroxychloroquine, azithromycin, lopinavir-ritonavir, ribavirin and interferon alfa-2a; in addition to methylprednisolone 80 mg daily and empiric antibacterial therapy. Lower respiratory cultures taken on day 18 yielded a growth *A. terreus* and *C. albicans*, but no bacterial pathogens. At the same time, there was evidence of progressive bilateral opacification in his chest radiograph and worsening respiratory failure. Voriconazole 400 mg 12 hourly was started on day 21. Over the subsequent 3 weeks, the patient’s condition remained critical and he developed multiple complications including acute renal failure, bilateral digital gangrene and sacral pressure sores. A chest x-ray taken on day 44 showed further progression of the previously noted bilateral opacification. On day 49, he developed a cardiopulmonary arrest and passed away. Post-mortem examination was not performed in either case reported here.

## Discussion

Both patients in this report had critical COVID-19, required invasive mechanical ventilation, and received broad-spectrum antibacterial therapy and high dose systemic corticosteroids. Both died with progressive respiratory failure.

Confirmation of CAPA, rather than airway colonization, is challenging for many reasons. Firstly, the interpretation of x-ray abnormalities is confounded by pre-existing viral pneumonia changes, and characteristic CT findings associated with invasive aspergillosis are rare in immune competent individuals.[[4], [5], [6]] Secondly, the diagnostic utility of serum galactomannan in immune competent hosts is unsatisfactory.[4,6] Biomarker and PCR assays for *Aspergillus species* have better performance on bronchoalveolar lavage (BAL). However, bronchoscopy is discouraged in patients with COVID-19 due to the associated aerosol generation and high risk of SARS-CoV-2 transmission to healthcare workers.[6,7]

The likelihood of CAPA is assessed on the basis of clinical, radiological and mycological data in individual patients. Widely accepted criteria for the diagnosis of putative invasive aspergillosis in critically ill patients include the isolation of *Aspergillus species* from lower respiratory tract cultures, in addition to any one of compatible symptoms (e.g.; fever, worsening respiratory failure), abnormal chest radiology, host risk factors (e.g.; neutropenia, high dose glucocorticoid therapy), or demonstration of branching hyphae on BAL smear.[5] Accordingly, both patients in this report had putative CAPA.

The rate of putative invasive aspergillosis in patients with critical COVID-19 is alarmingly high in some reports. For example, CAPA was reported in 8 (29.6%) out of 27 consecutive critical COVID-19 cases from France, 5 (26.3%) out of 19 critical COVID-19 from Germany, 7 (20.6%) out of 34 cases from the Netherlands, and 5 (21.7%) out of 23 cases in Pakistan.[[8], [9], [10], [11]] Similar to the cases described here, patients reported with CAPA were mostly immune competent, had radiological abnormalities typical of COVID-19, and many received systemic steroids as part of their COVID-19 care.[[8], [9], [10], [11], [12], [13], [14], [15]] However, whereas bronchoscopy was not performed in our patients due to their critical status and for infection control considerations, many reported cases were diagnosed on the basis of cultures, galactomannan detection or PCR on BAL samples.[[8], [9], [10],^12^,^14^,^15^] Lung biopsy is generally considered risky in ventilated patients, though this has occasionally been performed in patients with suspected CAPA.[5,10] Post-mortem histopathological confirmation of CAPA has also been reported.[16]

Like influenza-associated invasive pulmonary aspergillosis, CAPA appears to be associated with high mortality. Out of 40 CAPA cases thus far reported in the literature, 25 (62.5%) have died (Table 1).[5,[8], [9], [10], [11], [12], [13], [14],[16], [17], [18], [19], [20]] Such high mortality is not surprising given that CAPA has been almost exclusively reported in critically ill COVID-19 requiring mechanical ventilation, who were mostly of older age and had significant co-existing chronic medical conditions. Delayed recognition and commencement of appropriate antifungal therapy may have also contributed to the poor outcomes associated with CAPA. Voriconazole and isavuconazole are generally considered first line treatment options for invasive aspergillosis, though supporting data is mainly derived from hematology settings.[21] Lipid formulations of amphotericin are appropriate alternatives if azoles are contra-indicated, and in settings with higher background prevalence of azole resistance in *Aspergillus species*.[17,18,21]

**Table 1:** Summary of previously reported cases of COVID-19-associated pulmonary aspergillosis.

Study	Country	CAPA*	Aspergillus species	Aspergillus species in culture	Serum or BAL GM positive	BAL Aspergillus PCR positive	Initial Antifungal Therapy	In-hospital mortality
Alanio, 2020 [8]	France	9/27	*A. fumigatus* (7/9)	7/9	3/9	4/9	VOR (1/9) CAS (1/9) None (7/9)	4/9
Koehler, 2020 [9]	Germany	5/19	*A. fumigatus* (5/5)	3/5	4/5	4/5	VOR (2/5) ISA (1/5) CAS (2/5)	3/5
Rutsaert, 2020 [10]	The Netherlands	7/34	*A. fumigatus* (5/7) *A. flavus* (1/7)	6/7	6/7	NR	VOR (4/7) VOR/ISA (2/7) None (1/7)	4/7
Nasir, 2020 [11]	Pakistan	5/23	*A. flavus* (3/5) *A. niger* (1/5) *A. fumigatus and A. flavus* (1/5)	5/5	0/5	NR	VOR (3/5) AmB-d (2/5)	3/5
van Arkel, 2020 [12]	The Netherlands	6/31	*A. fumigatus* (6/6)	5/6	3/6	NR	VOR plus AND (5/6) L-AmB (1/6)	4/6
Blaize, 2020 [13]	France	1/1	*A. fumigatus*	1/1	0/1	1/1	None	1/1
Lahmer, 2020 [14]	Germany	2/2	*A. fumigatus* (2/2)	2/2	2/2	NR	L-AmB (2/2)	2/2
Wang, 2020 [15]	China	8/104	*A. fumigatus*	8/8	NR	NR	NR	NR
Antinori, 2020 [16]	Italy	1/1	*A. fumigatus*	1/1	1/1	1/1	L-AmB	1/1
Ferreira, 2020 [17]	France	1/1	*A. fumigatus*	1/1	0/1	1/1	None	1/1
Meijer, 2020 [18]	France	1/1	*A. fumigatus*	1/1	1/1	NR	VOR plus CAS	1/1
Prattes, 2020 [19]	Austria	1/1	*A. fumigatus*	1/1	0/1	NR	VOR	1/1
Sharma, 2020 [20]	Australia	1/1	*A. fumigatus*	1/1	NR	NR	VOR	0/1

*number with CAPA/total number in the report

AmB-d, amphotericin b deoxycholate; AND, anidulafungin; BAL, bronchoalveolar lavage; CAPA, COVID-19-associated pulmonary aspergillosis; CAS, caspofungin; GM, galactomannan; ISA, isavuconazole; L-AmB, liposomal amphotericin; NR, not reported; VOR, voriconazole

In our report, putative CPAP cases were caused by *A. terreus* or *A. niger.* Unlike Europe and North America, non-*fumigatus Aspergillus species* are more common in the Middle East and South Asia.[22] Interestingly, this geographic variation in *Aspergillus species* distribution is also notable in the previous CPAP report from Pakistan, compared with reports from other parts of the world (Table 1).[5,[8], [9], [10], [11], [12], [13], [14],[16], [17], [18], [19], [20]].

In conclusion, CAPA should be considered in patients with critical COVID-19 who have unexplained progressive respiratory failure despite optimized supportive care. Depending on locally available diagnostic resources, tracheal aspirates, or preferably BAL if feasible, should be sent for fungal cultures, galactomannan, and Aspergillus PCR. Empiric systemic antifungal therapy may be justified in selected cases, pending diagnostic work up results. Large, multi-center studies are required to further understand the pathogenesis of invasive aspergillosis in COVID-19, and the optimal diagnostic and treatment strategies.

## Ethics statement

The report was approved by the Institutional Review Board (MRC0120191) with a waiver of informed consent.

## Declaration of Competing Interest

The authors do not have an association that might pose a conflict of interest in relation to this work.

## Funding

The publication of this article was funded by the Qatar National Library.

## CRediT authorship contribution statement

**Shiema Abdalla:** Conceptualization, Data curation. **Muna A. Almaslamani:** Data curation. **Samar M. Hashim:** Writing - review & editing. **Abdulsalam S. Ibrahim:** Writing - review & editing. **Ali S. Omrani:** Conceptualization, Supervision.
